# Microstructural Evolution of Quaternary AlCoCrNi High-Entropy Alloys during Heat Treatment

**DOI:** 10.3390/ma17143617

**Published:** 2024-07-22

**Authors:** Elyorjon Jumaev, Hae-Jin Park, Muhammad Aoun Abbas, Dilshodbek Yusupov, Sung-Hwan Hong, Ki-Buem Kim

**Affiliations:** 1 FIE UzLITI Engineering LLC, 28B Beshyogoch, Tashkent 100066, Uzbekistan; elyorjon.jumaev@gmail.com; 2 Department of Nanotechnology and Advanced Materials Engineering, Sejong University, 209, Neungdong-ro, Gwangjin-gu, Seoul 05006, Republic of Korea; haejinp@sejong.ac.kr (H.-J.P.); m.aoun577@gmail.com (M.A.A.); dilshodbek.yusupov5260@gmail.com (D.Y.); shhong@sejong.ac.kr (S.-H.H.)

**Keywords:** high-entropy alloy, mechanical properties, chemical composition, heat treatment, sigma phase

## Abstract

This study examines the microstructural evolution and mechanical properties of quaternary AlCoCrNi high-entropy alloys after heat treatment at 873 K for 72 and 192 h. The changes in nanostructure and phase transformation based on the heat treatment duration were as follows: B2 dendrite + BCC interdendrite and sigma phases after 72 h; B2 dendrite and interdendritic sigma phases + BCC after 192 h. After annealing, the morphology of the dendritic region shifted from spherical to needle-like, and the interdendritic region transformed from a spinodal-like to a plate-like morphology. Additionally, a phase transformation was observed in the dendritic regions of the annealed alloys at the nano-scale. The presence of the sigma phase in AlCoCrNi high-entropy alloys significantly improved the yield strength to around 1172 MPa; nevertheless, it decreased the compressive strain rapidly to 0.62%.

## 1. Introduction

Currently, high-entropy alloys (HEAs) are considered a new division of metallic materials. An HEA comprises five or more major components with equiatomic or near-equiatomic ratios [1,2,3,4]. This alloy system has received a considerable amount of interest from researchers because of its superlative wear and corrosion resistance, high hardness, thermal stability, and thermal softening resistance [5,6,7]. HEAs have high configurational entropy because of their equimolar or near-equimolar structure that enhances the solid-solution forming phases [8,9,10,11,12,13,14,15,16,17]. As a consequence, most HEAs form solid-solution phases with basic components, such as face-centered cubic (FCC) and body-centered cubic (BCC) structures [18,19]. 

Furthermore, when the alloys are heat-treated at >873 K, the BCC phases from an as-cast solution can be intermetallic, as reported [7,19,20,21]. The minor elements and the heat treatment process have a significant influence on the formation of the intermetallic phases and the mechanical behaviors of HEAs. Specifically, the sigma phase formation is generally reported in heat-treated HEAs. The sigma phase has high strength and hardness but limited ductility; therefore, its development contributes to major variations in mechanical characteristics. Controlling the amount and the sigma phase formation is important for the creation of HEAs according to the required applications [22,23,24,25,26,27,28,29]. In addition, the sigma phase formation enhances the characteristics of HEAs [20]. Nevertheless, the intrinsic existence of the sigma phase further complicates this endeavor. Structural materials that contain Co and Cr, which include Ni-based superalloys and stainless steels, present brittleness with high-temperature applications because of the sigma phase [29]. Moreover, the sigma phase in HEAs can also contain many components, which may impact their stable phase temperature to increase or decrease from 873 K depending on the prolonged heat treatment time [23,24,25,26,27,28,29,30,31,32].

Generally, the involved elements depict the range of the valence electron concentration (VEC) that may form the sigma phase. Initially, the sigma phase is predicted in the VEC range, relying on the following elements: Al, Co, Cr, Cu, Fe, Mn, Ni, Ti, and V. *d*-Shell elements have an obvious, high impact on the formation of the sigma phase in the VEC range [29]. Additionally, the possibility of the sigma phase cannot be completely eliminated in the alloys (VEC < 7.85) [29,30,31,32]. These factors demonstrate that subsequent studies involving a number of alloy compounds and heat treatment temperatures are required to develop a deeper understanding of the sigma phase formation in HEAs.

The microstructure of as-cast AlCoCrNi HEAs has been studied in the literature, demonstrating that the hardness of AlCoCrNi HEA alloys decreases significantly at heat treatments at >873 K [33]. Moreover, a detailed discussion about the phase transformation at 873 K is lacking; therefore, we investigated the phase transformation and the morphological changes at the nano-scale after heat treatment at 873 K for periods of 72 h and 192 h. In addition, the specific yield strength of HEAs and the thermodynamic aspects are discussed, comparing them with those of previous HEAs, which may promote the development of HEAs.

## 2. Materials and Methods

The AlCoCrNi HEAs were prepared using a vacuum arc-casting method under an argon atmosphere. The samples comprising more than 99% high-purity elements were remelted five times to obtain chemical homogeneity, and the last step was a suction casting process, where the cylindrical rod-type samples, 3 mm in diameter and 50 mm in length, were prepared in a water-cooled copper mold. The alloys were heated at 873 K for different time periods, i.e., 72 and 192 h in a vacuum furnace. The samples were analyzed to investigate the microstructure and the phase evolution of the HEAs by using X-ray diffraction (XRD) with CuKα radiation (Rikagu-D/MAX-2500/PC, Tokyo, Japan), scanning electron microscopy (SEM; JSM-6390, JEOL, Tokyo, Japan), and field emission scanning electron microscopy (FE-SEM; SU8010, Hitachi, Tokyo, Japan). Transmission electron microscopy (TEM; Technai F20, Hillsboro, OR, USA) with energy-dispersive spectrometry (EDS) was used to analyze the phase formation and the crystal structure. The TEM ingots were prepared using ion milling with liquid nitrogen cooling. For the HEA compression test, rod-shaped samples were made with a 2:1 side ratio between length (6 mm) and diameter (3 mm) and examined using a universal measurement device at a strain rate of 1 × 10^−3^ s^−1^ (UTM; Zwick Roell Z050, Ulm, Germany). The high-range temperature test for hardness was performed using a 120° diamond indenter with a 1471 N load at temperatures that ranged from 293 to 1173 K.

## 3. Results and Discussion

**Figure 1:**
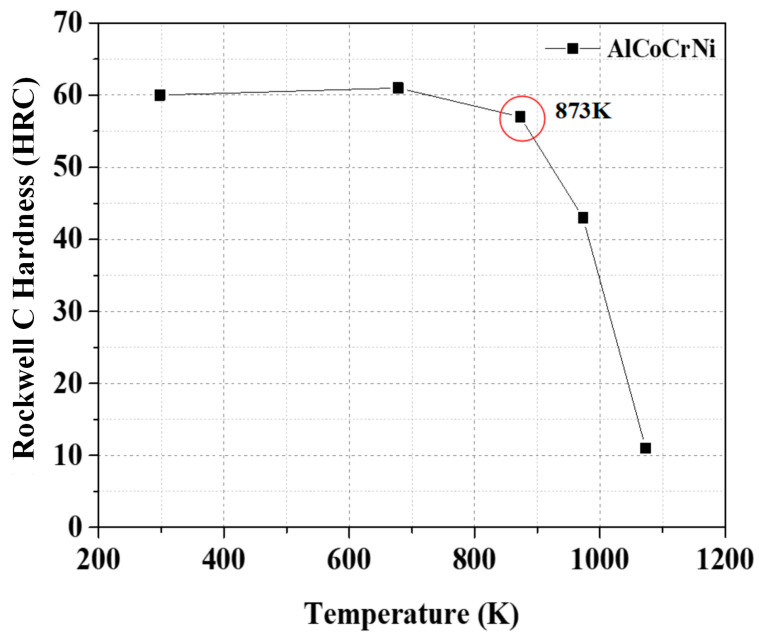
High-temperature hardness test results of the as-cast and heat-treated AlCoCrNi HEAs; the red circle indicate the hardness at 873 K.

To specify the appropriate homogenization heat treatment of HEAs, a comprehensive understanding of the phase transformation temperatures and, in particular, the liquidus temperatures of alloys is essential. Rockwell C hardness at different temperatures was used to measure the effect of temperature on the mechanical behavior of the as-cast AlCoCrNi HEA. Figure 1 illustrates the Rockwell C hardness values of as-cast AlCoCrNi HEAs at temperatures that ranged from 297 to 1073 K. A significant decrease occurs in the alloy’s hardness at 873 K [33]. Since studies have shown that heating these alloys at 873 K may cause a phase transformation, the AlCoCrNi HEAs were heat-treated for further analysis of the morphology and phase transformation at 873 K for 72 and 192 h.

**Figure 2:**
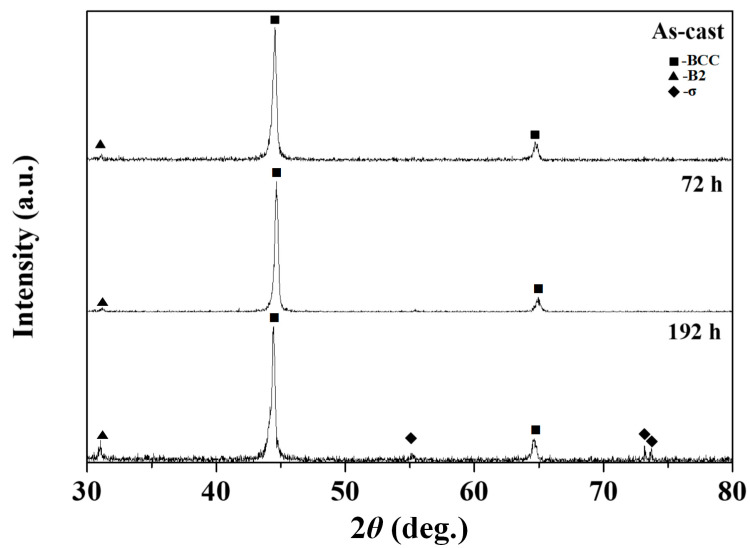
XRD patterns of as-cast and heat-treated AlCoCrNi HEAs at 873 K for 72 and 192 h.

Figure 2 presents the XRD patterns of the as-cast and heat-treated AlCoCrNi HEAs at 873 K for 72 and 192 h, showing several diffraction peaks between 2*θ* = 30 and 80 degrees, which correspond to the high-intensity peaks of the BCC and the low-intensity peak of the B2 phases, respectively. For the 192 h heat-treated sample, it was observed that the intensity of the XRD peak at approximately 32 degrees 2θ corresponding to the B2 phase is revealed more distinctly. It indicates that the alloy undergoes atomic ordering over time, resulting in the stabilization of the B2 phase. Such ordering typically occurs at elevated temperatures, where diffusion processes are active, allowing for atoms to rearrange into a more energetically favorable ordered state. Meanwhile, the B2 phase can enhance hardness and strength; it may also introduce brittleness due to the limited slip systems available in the ordered structure.

Nevertheless, low-intensity signals of the sigma (σ) phase were observed in the alloys annealed at 873 K for 192 h. In addition, the XRD patterns showed that during the annealing for 72 h, no noticeable changes occurred, whereas the sigma phase appeared during the long-time heat treatment for 192 h.

**Figure 3:**
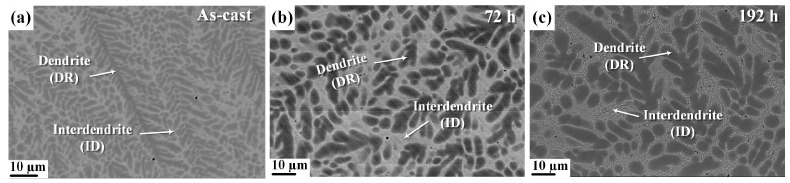
SEM backscattered electron images illustrating the microstructures of the (**a**) as-cast and (**b**) heat-treated HEAs at 873 K for 72 h and (**c**) 192 h.

Figure 3a–c illustrate the backscattered SEM images of the as-cast and annealed AlCoCrNi HEAs, showing relatively dark dendritic regions and bright interdendritic regions. Figure 3b,c show that the heat treatment results in an increased volume fraction of the interdendrite and a decreased volume fraction of the dendrite, as summarized in Table 1. In previous studies, the volume fraction of as-cast AlCoCrNi was calculated to be 59.6% dendrite and 40.4% interdendrite [33]. In the current study, 53.1% dendrite and 46.9% interdendrite were present in the alloy that was heat-treated for 72 h, whereas 50.9% dendrite and 49.1% interdendrite were in the alloy that was heat-treated for 192 h. 

The volume fraction difference indicates that the interdendrite’s BCC phase expanded under the influence of the heat treatment; however, the long-term heat treatment resulted in a small precipitate formation in the interdendritic region, as shown in Figure 3b. 

To understand the nature of the dendritic and interdendritic regions of HEAs, we performed an EDS analysis of each region, as shown in Table 2. The as-cast alloy, as analyzed in [33], has a chemical composition in the dendritic region (DR) rich in Al (28.4%) and Ni (30.3%), whereas the interdendritic region (ID) is slightly enriched with Co (26.7%) and Cr (31.7%) [33]. 

In the heat-treated alloys, the EDS results show noticeable differences. For example, the amount of Al and Ni slightly decreased from 28.4% to 24.6% and 30.3% to 26.9%, respectively, in the dendritic region when prolonging the time of the heat treatment, whereas the amount of Co and Cr increased from 25.3% to 29.2% and 16% to 19.3%, respectively, as shown in Table 2. For the composition in the interdendritic region, the amount of Al and Ni increased from 18.4% to 22.3% and 23.2% to 24.5%, respectively, whereas the amount of Co and Cr decreased from 26.7% to 24.3% and 31.7% to 28.9%, respectively.

**Figure 4:**
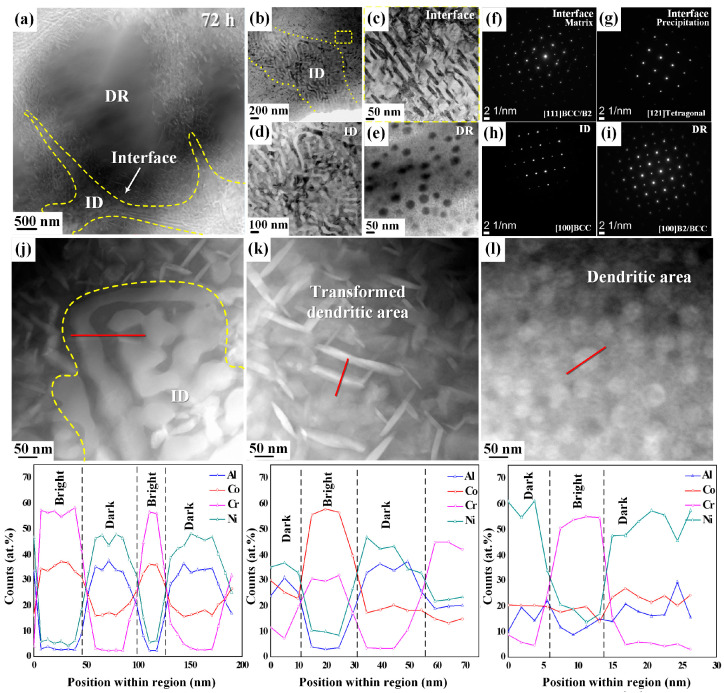
(**a**) The STEM HAADF image with dendritic (DR) and interdendritic (ID) regions distinguished by yellow dashed lines; (**b**) the high-magnification bright-field image; (**c**) the bright-field image corresponding to the interface region taken from the yellow rectangle in Figure 4b; (**d**,**e**) bright-field images corresponding to the dendritic and interdendritic regions; (**f**–**i**) nano-beam diffraction and the SAED corresponding to the bright area of the interface and the needle-like phase with dark areas of the interface, interdendritic (ID), and dendritic (DR) regions; (**j**–**l**) STEM HAADF images and EDS elemental graphs (regions indicated by red line) of the AlCoCrNi HEAs heat-treated at 873 K for 72 h.

We conducted a detailed investigation of the phase transformation using a TEM analysis. Figure 4 illustrates the nano-scale morphology and phase formation of AlCoCrNi HEAs annealed at 873 K for 72 h. The dendritic region exhibits spherical precipitates, whereas a spinodal-like nanostructure is present in the interdendritic region, as revealed in the high-angle annular dark-field (HAADF) and high-angle annular bright-field (HAABF) images in Figure 4a,b. Morphological changes occurred in the dendritic and interdendritic regions, as shown in Figure 4a. Under high magnification, a yellow dotted line separates the regions; an area of their interface is highlighted as a rectangle in Figure 4b. In addition, the magnified images of both the dendrite and interdendrite regions shown in Figure 4a can be found in Figure 4d and 4e, respectively. These images confirm the presence of a dual structure within these regions. 

The interface shows the presence of a needle-like structure in Figure 4c, and its nano-beam diffraction (NBD) pattern is shown in Figure 4f,g. In Figure 4f, the NBD pattern shows the BCC/B2 phases in the bright region (matrix), whereas the sigma phase, which is a tetragonal crystal structure, is observed with the [121] zone axis in a needle-like structure, as shown in Figure 4g. The interdendritic regions show a spinodal-like morphology with diffraction spots that corresponds to the BCC-A2 phase along the [100] zone, as shown in Figure 4h. 

Meanwhile, spherical precipitates are present in the dendritic portion, and the A2 phase with a superlattice of the B2 phase is visible in the SAED (selected area electron diffraction) pattern recorded along the [100] zone of the BCC-B2 phase, as shown in Figure 4i. 

These results show that both the dendritic and interdendritic regions contain an ordered B2-BCC phase and a disordered A2-BCC phase. TEM with EDS was used to measure the chemical composition, as listed in Table 3, which was compared with the as-cast AlCoCrNi HEAs [33]. The chemical composition of the alloys was examined using the TEM-EDS shown in Figure 4j,l’s dark-field STEM (scanning transmission electron microscopy) images, as summarized in Table 3. The TEM-EDS results suggest that the dendritic, interdendritic, and interface regions exhibit a Co-Cr-rich bright contrast and Al-Ni-rich dark contrast; however, a high amount of Co (55.7%) was found in the bright precipitates of the interface region where the sigma phase was observed.

**Figure 5:**
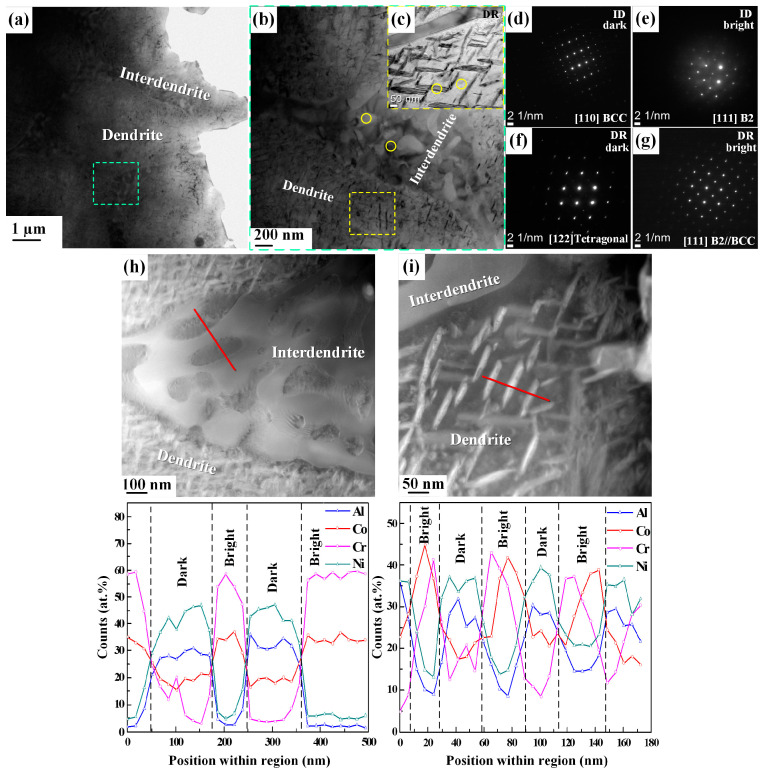
(**a**) Bright-field STEM images; (**b**) bright-field STEM images taken from the green rectangle in (**a**); (**c**) the bright-field image corresponding to the dendritic region taken from the yellow rectangle in (**b**); (**d**,**e**) nano-beam diffractions corresponding to the dark and bright areas of the interdendritic region; (**f**,**g**) nano-beam diffractions corresponding to the dark and bright areas of the dendritic region; and (**h**,**i**) STEM HAADF images and EDS elemental graphs (regions indicated by the red line) of the AlCoCrNi HEA heat-treated at 873 K for 72 h.

Figure 5a shows a bright-field TEM image taken from the sample heat-treated at 873 K for 192 h, identifying the dendritic and interdendritic regions. A spinodal-like morphology is observed in the interdendritic region, as shown in Figure 5b. Additionally, the TEM analysis illustrates a plate-like unique morphology in the dendrite area, as shown in Figure 5c. The dendritic region morphology is totally transformed from a spherical shape to a plate-like structure. 

Furthermore, the NBD images taken from both areas are given in Figure 5d–g. Figure 5d,e present the BCC and B2 crystal lattice in the dark and bright portions of the interdendritic region. In Figure 5f, the tetragonal crystal structure, which is the sigma phase, is found in the dark area of the dendritic region. Nevertheless, a B2/BCC crystal lattice is observed in the bright matrix area of the dendritic region in Figure 5g. 

The composition of the alloy was determined using TEM with EDS, as shown in Figure 5h,i and summarized in Table 3. The as-cast sample demonstrates both bright Cr-rich and dark Ni-rich areas of the dendritic and interdendritic regions. Co is equally identified in both regions [33]. The nano-scale EDS analysis of the heat-treated sample reveals that the elements are distributed accordingly throughout the regions, including Al and Ni in the dark areas and Co and Cr in the bright areas of the dendritic regions, as shown in Figure 5i and Table 3, and Figure 5h shows the interdendritic regions. 

These results indicate that after heat treatment at 873 K for 192 h, Co behaved differently in the dark and bright areas of the interdendritic and dendritic regions compared to the behavior in the as-cast alloy. Additionally, by increasing the annealing period, the amount of Cr and Co in the dendritic region also increases, as evidenced by the sigma phase formation, the probability of which is high in most Co-Cr-rich alloys [34]. These observations show that a long heat treatment causes the development of the sigma phase.

**Figure 6:**
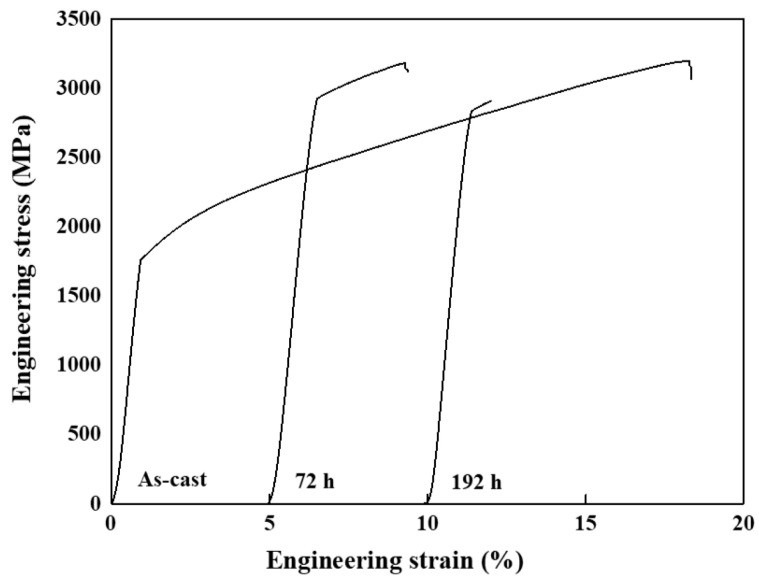
Engineering compressive stress–strain curves for the AlCoCrNi HEAs as-cast and heat-treated at 873 K for 72 and 192 h.

The results of the engineering compressive stress and strain of the as-cast and annealed AlCoCrNi HEAs are represented in Figure 6 and summarized in Table 4. The as-cast alloy presents about a 16.71% compressive strain and a 1753 MPa compressive stress [33]. The compression test results of the heat-treated HEAs at 873 K for 72 and 192 h reveal that the yield strength significantly increased, whereas a decrease occurred in the compressive strain of the alloy. 

The most pronounced effect on the yield strength was observed in the sample that was heat-treated for 72 h because of the alternative morphology of the sigma and B2 phases. This heat-treated sample showed the highest yield strength compared to the as-cast samples, with an almost 1172 MPa difference between the as-cast and 72-h heat-treated samples. However, a high amount of the sigma phase reduces the yield strength and strain, as shown in the results when the sample was heat-treated at 873 K for 192 h, which yielded an engineering stress of about 2834 MPa and a strain of about 0.62%.

As the long heat treatment assisted atomic diffusion more sufficiently, the Co and Cr atoms in the interdendritic region moved into the dendritic region because of their negative mixing enthalpy. The TEM with EDS results in Table 3 indicate that the atomic ratio of the Co and Cr decreased in the interdendritic region and increased in the dendritic region with a longer heat treatment. In the dendritic region, the rise in Cr with a large atomic radius produced significant lattice distortion, whereas the precipitation of some particles discharged the lattice distortion energy that enhanced the strength of the alloy. However, the decreased Co-Cr content released the lattice distortion in the interdendritic region, so the interdendritic region could keep the Al-Ni-rich solid-solution state. On the other hand, an incomplete transformation of the Co-Cr from the interdendritic region to the dendritic region led to the sigma phase formation at the interface in the alloy heat-treated for 72 h. Moreover, the sigma phase was observed with the B2 and BCC-A2 phases. Therefore, this alloy presents high strength and strain compared to the alloy heat-treated for 192 h, which shows a complete transfer of the Co and Cr and results in a higher concentration of the sigma phase in the dendritic area.

In addition to the thermodynamic computations, the VEC has been suggested for the phase stability predictions as a possible parameter [29,30]. The experimental findings are used to describe the empirical VEC ranges of the stable phase combinations. This method cannot provide accurate correlations across a number of various alloy systems, but it can be beneficial within an alloy family [29,30,31,35,36]. The definition was explicitly extended to predict the formation of the sigma phase [30,35], where an alloy that consists of Cr as a major element with a VEC between 6.88 and 7.84 is likely subjected to the formation of the sigma phase at 873 K. The VEC for the AlCoCrNi alloy is 7.0, calculated using the following equation:

$$\mathrm{VEC}=\sum_{i=1}^{n}C_{i}{\left(VEC\right)}_{i}$$

where *C_i_* is the atomic concentration and *(VEC)_i_* is the VEC for the *i_th_* element, respectively.

To better understand the validity of the VEC concept and phase stability of the heat-treated samples obtained, we analyzed findings from the literature summarized in Table 5 [30,31,33,35,36]. Additionally, at 873 K, the VEC method forecasts the propensity of the AlCoCrNi alloy to develop the sigma phase. The experimental results also implied the sigma phase formation would occur in the equiatomic HEAs after heat treatment [30,35]. 

Nevertheless, this approach is restricted in its implementation because of the critical VEC range where the sigma phase is likely to develop. Its application depends on temperature, and thus, many experimental data points are necessary to define these critical ranges at different times and temperatures. 

In conclusion, the first principle is provided for predicting the sigma phase presence in HEAs. Alloys that have VEC values of 6.88–7.84 are inclined to form the sigma phase in the as-cast alloys or at appropriate temperatures during heat treatment. The factors are well extended to HEAs consisting of Cr or Co. The prediction of the formation of sigma phases is essential to the design of HEAs.

## 4. Conclusions

This study analyzed the effect of the heat treatment on a nano-scale structure and the mechanical properties of the quaternary AlCoCrNi HEAs. The heat-treated HEAs illustrate a unique plate-like morphology. The compression test presented an increase in yield strength but a decrease in compressive strain of the heat-treated HEAs despite increasing the heat treatment time. According to the TEM analysis and the compression test results, the following conclusions are drawn:

1. The highest yield strength for the HEAs was determined to be approximately 2925 MPa in the sample heat-treated at 873 K for 72 h, and remarkable variations in the nano-scale morphology compared to the as-cast alloy were observed.
2. When the alloy was heat-treated at 873 K for 72 h, an interface with the sigma phase developed between the dendritic and interdendritic regions.
3. In comparison, in the alloy heat-treated at 873 K for 192 h, the morphology of the dendritic area completely transformed from spherical to plate-like, and a tetragonal crystal structure developed, which is the sigma phase. At the same time, the engineering strain of the HEAs also reduced with an increase in the sigma phase.
4. The formation of the sigma phase due to varying heat treatment durations can affect the mechanical properties, and prolonged heat treatment may result in a diminished strengthening effect on the alloy.
5. Prolonged annealing or extended heat treatment plays a crucial role in the microstructural evolution and phase stability of HEAs. This process can lead to significant phase transformations, resulting in the formation of ordered structures and secondary phases. Further studies are necessary to induce similar phase transformations, balancing improved strength and plasticity with the potential brittleness caused by the sigma phase.

## Figures and Tables

**Table 1:** The volume fraction of the micro-scale dendritic and interdendritic regions of the as-cast and heat-treated AlCoCrNi HEAs calculated from the SEM-BSE (backscattered electrons) images.

Alloy	Dendrite Volume Fraction (%)	Interdendrite Volume Fraction (%)
As-cast [33]	59.6	40.4
Heat-treated for 72 h	53.1	46.9
Heat-treated for 192 h	50.9	49.1

**Table 2:** The chemical composition of the micro-scale dendritic and interdendritic regions of the as-cast and heat-treated AlCoCrNi HEAs measured using the FE-SEM EDS analysis.

Region	Element Composition (%)			
	Al	Co	Cr	Ni
As-cast [33]				
Dendrite	28.4	25.3	16	30.3
Interdendrite	18.4	26.7	31.7	23.2
Heat-treated sample (at 873 K for 72 h)				
Dendrite	26.3	27.6	17.7	28.4
Interdendrite	20.8	25.5	30.2	23.5
Heat-treated sample (at 873 K for 192 h)				
Dendrite	24.6	29.2	19.3	26.9
Interdendrite	22.3	24.3	28.9	24.5

**Table 3:** Chemical composition of nano-grained A2 and B2 phases in the dendritic and interdendritic regions of the as-cast and heat-treated AlCoCrNi HEAs measured using the TEM with EDS analysis.

Region	Brightness	Element Composition (%)			
		Al	Co	Cr	Ni
As-cast [33]					
Dendrite	Dark	23.2	28.3	9.2	39.3
	Bright	8.9	28.9	48.2	14
Interdendrite	Dark	20.3	27.3	15.2	37.4
	Bright	3.1	30.7	60.6	5.6
Heat-treated sample (at 873 K for 72 h)					
Dendrite	Dark	18.7	20.3	7.8	53.7
	Bright	9.8	21.1	49.5	19.6
Interface	Dark	24.6	25.3	9.6	40.5
	Bright	3.8	55.7	30.2	10.3
Interdendrite	Dark	33.4	16.2	2.8	47.6
	Bright	2.9	34.5	56.9	5.7
Heat-treated sample (at 873 K for 192 h)					
Dendrite	Dark	28.9	18.7	16.3	36.1
	Bright	9.2	36.7	40.8	13.3
Interdendrite	Dark	30.1	19.4	6.8	43.7
	Bright	8.4	30.5	44.9	16.2

**Table 4:** The yield strength *σ_y_* and compressive strain *ε_p_* of the as-cast and heat-treated AlCoCrNi HEAs determined by the compression.

Alloy	Compressive Yield Stress [MPa]	Compressive Strain (%)
As-cast [33]	1753	16.71
Heat-treated for 72 h	2925	2.78
Heat-treated for 192 h	2834	0.62

**Table 5:** The presence of σ in the heat-treated HEAs collected from the previous studies.

Alloys	VEC	Phase	Heat-Treatment Condition
AlCoCrNi	7.0	B2 + BCC + σ	873 K 72, 192 h
AlCoCeFeNi [30]	7.2	B2 + FCC + σ	973 K, 20 h
Al_0.3_CrFe_1.5_MnNi_0.5_ [23]	7.19	B2 + FCC + σ	973 K, 2 h
Al_5_Cr_32_Fe_35_Ni_22_Ti_6_ [24]	7.31	B2 + FCC + σ	973 K, 20 h
CoCrCuFeMnNiTiV [27]	8.5	B2 + FCC + σ	973 K, 20 h
Al_0.5_CoCrCuFeNiV [28]	7.77	B2 + FCC + σ	973 K, 20 h
AlCoCrCuFeNi [32]	7.83	B2 + FCC + σ	973 K, 20 h
Cr_2_CuFe_2_MnNi [30]	8.0	FCC + σ	973 K, 20 h
Al_0.5_CoCrCuFeNi [27]	8.27	FCC + σ	973 K, 24 h

## Data Availability

Data are contained within the article.
